# Alteration of Postural Reactions in Rats with Different Levels of Dopamine Depletion

**DOI:** 10.3390/biomedicines11071958

**Published:** 2023-07-11

**Authors:** Daria S. Kalinina, Vsevolod A. Lyakhovetskii, Oleg V. Gorskii, Polina Yu. Shkorbatova, Natalia V. Pavlova, Elena Yu. Bazhenova, Yurii I. Sysoev, Raul R. Gainetdinov, Pavel E. Musienko

**Affiliations:** 1 Institute of Translational Biomedicine, St. Petersburg State University Hospital, St. Petersburg State University, 199034 St. Petersburg, Russia; d.kalinina@spbu.ru (D.S.K.); gorskijoleg@gmail.com (O.V.G.); dakmin7@gmail.com (N.V.P.); bazhelen@mail.ru (E.Y.B.); gainetdinov.raul@gmail.com (R.R.G.); 2 Sechenov Institute of Evolutionary Physiology and Biochemistry, Russian Academy of Sciences, 194223 St. Petersburg, Russia; 3 Department of Neuroscience, Sirius University of Science and Technology, 354340 Sirius, Russia; 4 Pavlov Institute of Physiology, Russian Academy of Sciences, 199034 St. Petersburg, Russia; 5 Center for Biomedical Engineering, National University of Science and Technology “MISIS”, 119049 Moscow, Russia; 6 Department of Pharmacology and Clinical Pharmacology, Saint Petersburg State Chemical and Pharmaceutical University, 197022 St. Petersburg, Russia; 7 Life Improvement by Future Technologies Center “LIFT”, 143025 Moscow, Russia

**Keywords:** dopamine, Parkinson’s disease, dopamine transporter knockout (DAT-KO) rats, postural balance

## Abstract

Dopamine (DA) is the critical neurotransmitter involved in the unconscious control of muscle tone and body posture. We evaluated the general motor capacities and muscle responses to postural disturbance in three conditions: normal DA level (wild-type rats, WT), mild DA deficiency (WT after administration of α-methyl-p-tyrosine—AMPT, that blocks DA synthesis), and severe DA depletion (DAT-KO rats after AMPT). The horizontal displacements in WT rats elicited a multi-component EMG corrective response in the flexor and extensor muscles. Similar to the gradual progression of DA-related diseases, we observed different degrees of bradykinesia, rigidity, and postural instability after AMPT. The mild DA deficiency impaired the initiation pattern of corrective responses, specifically delaying the extensor muscles’ activity ipsilaterally to displacement direction and earlier extensor activity from the opposite side. DA depletion in DAT-KO rats after AMPT elicited tremors, general stiffness, and akinesia, and caused earlier response to horizontal displacements in the coactivated flexor and extensor muscles bilaterally. The data obtained show the specific role of DA in postural reactions and suggest that this experimental approach can be used to investigate sensorimotor control in different dopamine-deficient states and to model DA-related diseases.

## 1. Introduction

The control of body posture and balance is provided by spinal reflex mechanisms activated and modulated by supraspinal descending signals [1,2]. Dopamine (DA) is one of the crucial neuromodulators of muscle tone distribution, body posture configuration, and coordination of multi-muscle activity during movements [3,4]. DA is closely involved in the formation of intended behavior, motivation processes, and participates in integral movement control [5]. A decrease in dopaminergic (DA-ergic) neurotransmission leads to a motor activity decline, a deterioration in the speed of motor reactions, rigidity, and postural instability, which are observed in Parkinson’s disease (PD) or parkinsonism and dystonia [6,7]. The most frequently used animal models of dopamine deficiency are based on the neurotoxin-induced lesion of the nigrostriatal DA system by l-methyl-4-phenyl-l,2,3,6-tetrahydropyridine (MPTP) and 6-hydroxydopamine (6-OHDA), but this approach has limits, as the damage remains permanent [8] and development of new models is still important.

The main regulator of intracellular and extracellular DA levels is the DA transporter (DAT), which provides its reuptake [9,10]. It has been shown that the lack of DAT in DAT-knockout (DAT-KO) rats leads to an ~eight-fold increase in basal extracellular DA levels [11]. However, it was shown previously [12] that the inhibition of tyrosine hydroxylase (TH) by alpha-methyl-p-tyrosine (AMPT) leads to blocking of catecholamine synthesis and reduction of the tissue DA level in DAT-KO mice (0.2% of normal level). This extreme deficiency of DA after AMPT leads to PD-related manifestations: severe akinesia, rigidity, tremor, and ptosis [12]. Our recent study showed that AMPT in DAT-KO rats caused similar effects [13,14].

In the present study, we described the akinetic phenotype and investigated the dopamine-dependent changes in posture stability in DAT-KO and wild-type (WT) rats following AMPT treatment, which was assessed by analysis of kinematics and EMG activity during standing before and after the lateral horizontal displacement of the supporting platform [15].

## 2. Materials and Methods

### 2.1. Animals

All procedures were performed under the guidelines established by the European Community Council (Directive 2010/63/EU of 22 September 2010), and animal protocols were approved by the Ethics Committee of St. Petersburg State University, St. Petersburg, Russia (approval number 131-03-6). Dopamine transporter knockout (DAT-KO, *n* = 4) and wild-type (WT, *n* = 4) male rats 3–5 months old were used in the experiments. DAT-KO rats were generated by SAGE Laboratories using zinc-finger nucleases (ZFN) technology [16,17] for the elimination of the DAT gene. Breeding and genotyping of DAT-KO and WT rats were performed as described previously [9]. Rats were housed numbering three to five per cage before the surgical procedures and individually after it. They were maintained under standard lab conditions (12 h light/dark cycle, 21 ± 1 °C and 40–70% humidity), with food and water provided ad libitum. All experiments were conducted during the light phase.

### 2.2. Surgical Procedure 

To record EMG responses, the stainless steel wire electrodes (AS632, Cooner Wire, Chatsworth, CA, USA) were implanted into the left flexor (*Tibialis anterior m*.-TA_L) and bilaterally to the extensors (*Gastrocnemius lateralis m*., GM_L for the left and GM_R for the right hindlimb) muscles were performed under isoflurane anesthesia. The small notch of insulation (0.5 mm) was removed on each wire to expose the conductor and form the electrodes. The wires were inserted into the muscle through a 23 G needle and positioned in the middle of the muscle in the most responsive part, which was identified by electrical stimulation (the “hot spot”), then the wires were fixed together with an Ethilon 4 suture at the entrance and exit from the muscle [18]. The EMG reference electrode was implanted subcutaneously in the right shoulder using the same wire. Connectors were fixed on the skull by 4 screws and covered with dental cement. All experiments were performed in freely moving rats after the 5–8 days recovery period.

### 2.3. Experimental Design

**Figure 1:**
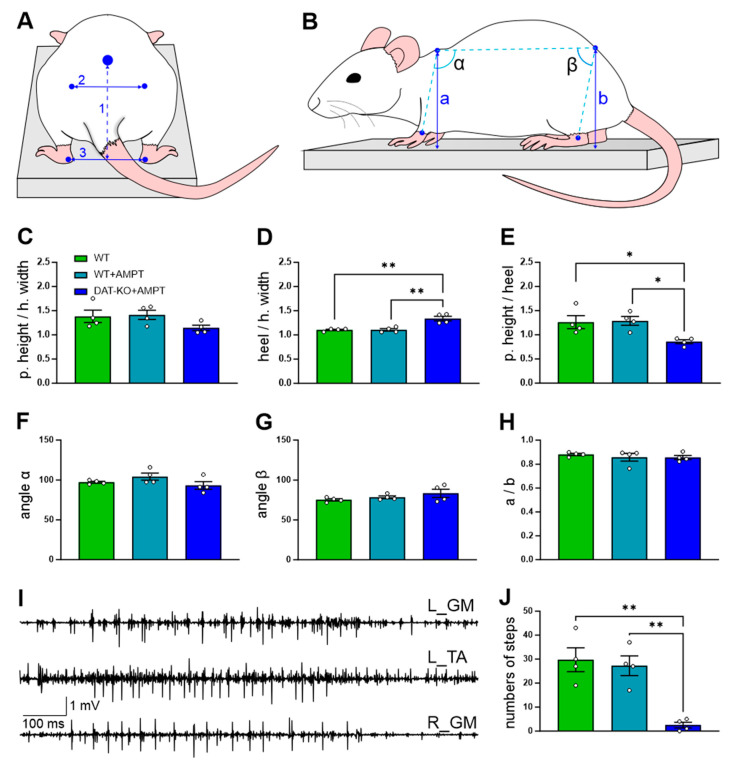
The general motor capacities of the rats with DA depletion. Rear-view (**A**) and side-view (**B**) of the body postural configuration during rat standing in three experimental conditions: WT, WT + AMPT, and DAT-KO + AMPT. 1—pelvic height; 2—hip width; 3—heel distance; a—withers height; b—pelvic height. (**C**) The ratio of the pelvic height to the hip width. (**D**) The ratio of the distance between heels to the hip width. (**E**) The ratio of pelvic height to the heel distance. (**F**) The mean angle between the carpal joint, withers and pelvis point (α in (**B**)). (**G**) The mean angle between withers, pelvis point, and ankle (β in (**B**)). (**H**) The ratio of the withers height to the pelvic height. (**I**) Example of tremor EMG for left hindlimb tibialis anterior muscle (TA_L), left (GM_L), and right (GM_R) gastrocnemius medialis muscles in standing DAT-KO + AMPT rats. (**J**) Numbers of steps during 90 s of “forced” walking in WT, WT + AMPT, and DAT-KO + AMPT rats. Significant differences between WT + AMPT and DAT-KO + AMPT in comparison by one-way ANOVA with post hoc Bonferroni’s multiple comparison test. Mean ± SEM. *—*p* < 0.05, **—*p* < 0.01.

To study DA-ergic control of postural function, three experimental groups were used: normal DA level (WT), mild DA deficiency (WT in 1 h after injection 250 mg/kg [14,19] of AMPT, WT + AMPT), and severe DA deficiency (DAT-KO after AMPT, DAT-KO + AMPT). Initially, we evaluated the common posture of the rat body. Identifying marks were drawn with a non-toxic marker on the shaved skin on the pelvis point (corresponding to the center of the pelvis), withers point (the ridge between the shoulder blades of an animal), and points on the trochanter major bilaterally. The pelvic height was measured as the distance between the pelvic point and the support surface. The hip width was measured as the distance between the trochanter major from the left and right. The ratios of different parameters were used for the actual evaluation of postural characteristics independently from anatomical differences caused by body size (Figure 1A,C–E): the ratio of the pelvic height to the hip width was used for the evaluation of the height of the pelvis above the platform, the distance between heels to the hip width–stance width, and pelvic height to the heel distance–dependence of the pelvis height from stance width. The angles between the carpal joint–withers–pelvis point (α in Figure 1B) and withers–pelvis point–ankle (β in Figure 1B), as well as the ratio of the withers height to the pelvic height, were used for comparison the rostrocaudal tilts of the body (Figure 1F–H). During the measurement, the rat was not restrained. We chose for analysis only those episodes in which the rat kept a suitable position (the head was straight and parallel to the surface, all 4 limbs were not mobile, and their position corresponded to a relaxed standing). To assess the disturbance of movement initiation after AMPT, we counted the number of steps during 90 s of “forced” walking (the rats were gently prompted to walk by the experimenter) in all groups. Horizontal lateral displacements were used for assessment of the dynamic postural stability and performed by the quick sliding of a narrow (8 cm) elevated walkway 2.5 cm to the left or to the right (regarding the rat), performed manually in the same manner while the animal stayed standing on all four limbs (Figure 1). The acceleration of the displacements was adjusted so as not to cause steps for the correction of postural balance (the maximum speed of displacements was 18–22 cm/s). Platform displacement parameters were controlled by a mechanical sensor—only similar displacements were selected for the analysis. Displacements were repeated unpredictably from 10 to 30 times for each rat. The EMG signals were differentially amplified (A-M Systems, Sequim, WA, USA, model 1700, bandwidth of 10 Hz–5 kHz), digitized at 20 kHz (LTR11, L-Card, Moscow, Russia), and further filtered in 100–2000 Hz bandwidth. In the WT group, EMG responses to the horizontal displacements were registered twice; that is, on the initial condition without any drugs and after intraperitoneal injection of 250 mg/kg AMPT in 1 h. 

### 2.4. Analysis and Statistics 

Custom scripts written in MATLAB (MathWorks, Inc., Natick, MA, USA) were used to analyze the EMG responses. Since pushes of the platform were performed manually, they took different amounts of time (216 ± 13 ms). For normalization, we did not use absolute time values, but rather bins for further analysis. We analyzed the amplitude distribution of the rectified EMG signal divided into 70 equal bins and normalized by integral muscle activity over these 70 bins. Bins from 30 to 40 corresponded to the lateral displacement of the platform. To verify that the rat had a quiet standing before and after the platform displacement, an epoch of three times the duration of the displacement was considered before (1–29 bins) and immediately after (41–70 bins) the displacement.

All statistical analyses were performed by using GraphPad Prism 9 (GraphPad Software, Inc., San Diego, CA, USA). All data are reported as mean ± standard error (SEM). The normal distribution of the results was checked by the Kolmogorov–Smirnov test. One-way ANOVA (ordinary for Figure 1 and repeated measures, RM, for others; F (DFn, DFd) were presented) with a post hoc Bonferroni multiple comparison test was used to assess the significance. The criterion level for the determination of statistical difference was set at *p* < 0.05.

## 3. Results

### 3.1. The General Motor Capacities of the Rats with DA Depletion

The body’s postural configuration of the DAT-KO rats was changed after the AMPT injection. Figure 1 shows the representative examples of the side views and rear views of the standing position in the three experimental conditions: WT, WT + AMPT, and DAT-KO + AMPT. Note that while standing, the DAT-KO + AMPT rats decreased tone in the extensors—the ratio of the pelvic height to the hip width (rear view, Figure 1A,C) had no significant differences (F(2, 9) = 2.264, *p* = 0.1598), but a tendency was observed (DAT-KO + AMPT: 1.15 ± 0.06 vs. WT: 1.4 ± 0.13 and WT + AMPT: 1.4 ± 0.13, *p* = 0.3580 and *p* = 0.2512, respectively). The ratio of the distance between heels to the hip width significantly differed between groups (F(2, 9) = 17.28, *p* = 0.0008) and in DAT-KO + AMPT is higher than in WT and WT + AMPT (1.4 ± 0.05 vs. 1.1 ± 0.01 and 1.1 ± 0.03, *p* = 0.0019 and *p* = 0.0020, respectively) that means that this group puts the hindlimbs wider (Figure 1D). The ratio of the pelvic height to heel distance (Figure 1E) significantly differed between the groups (F(2, 9) = 6.215, *p* = 0.0202), and in the rats with DA depletion was less than in other groups (DAT = KO + AMPT 0.86 ± 0.04 vs. WT 1.3 ± 0.13 and WT + AMPT 1.3 ± 0.09, *p* = 0.0485 and *p* = 0.0355, respectively), that also means large stance width in DAT-KO + AMPT. The rostrocaudal tilt of the body (side-view, Figure 1B,F–H) in rats of all three groups did not differ, as can be seen from the measurements of the angles between the carpal joint, withers and pelvis point (α) and between the withers, pelvis point, and ankle (β), as well as from the ratio of the withers height to the pelvic height.

DAT-KO rats after AMPT also had episodes of tremors while standing on the platform (Figure 1I), whereas other groups did not (Figure S1 of Supplementary Materials). The WT + AMPT rats demonstrated mild movement initiation disturbance, but the sum number of steps during 90 s (Figure 1J) did not show a significant difference compared to WT (post hoc *p* > 0.9999) while DAT-KO + AMPT due to progressive loss of the locomotor activity could make only a few steps (F (2, 9) = 15.81, *p* = 0.0011, DAT = KO + AMPT 2.5 ± 1.2 vs. WT 29.75 ± 5 and WT + AMPT 27.25 ± 4.1, with post hoc *p* = 0.0020 and *p* = 0.0038 respectively).

### 3.2. Postural Reactions of WT Rats to Lateral Platform Displacement

**Figure 2:**
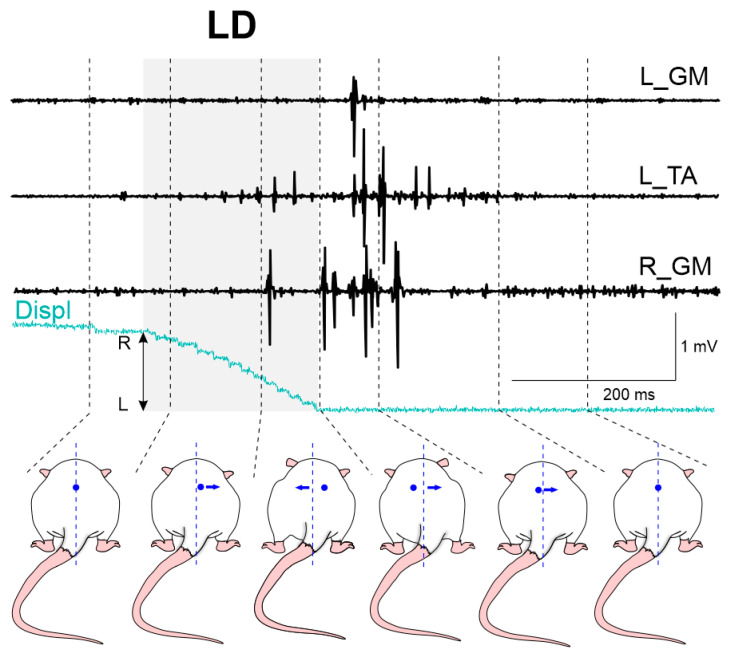
EMG activity of the left hindlimb tibialis anterior muscle (TA_L), the left (GM_L) and right (GM_R) gastrocnemius medialis muscles, and the body shift of WT rat during the leftward displacement (LD) of the platform. Displ—platform displacement sensor.

An example of a postural reaction to leftward displacement (LD) is presented in Figure 2. During LD. the rat’s body deviated to the right, relative to the platform; then, the platform slowed down and the torso inertially deviated to the left and the first peaks of EMG activity appeared, which was associated with the action of the left flexor TA and the right extensor GM. Further, after the platform stopped, the rat’s body deflected to the right (returning to the starting position), and a second peak of the TA flexor, possibly associated with muscle stretching, and also a peak of the left GM, which provided extension of the left limb being observed, while the right limb stabilized the posture, as suggested by the second peak of the right extensor GM.

**Figure 3:**
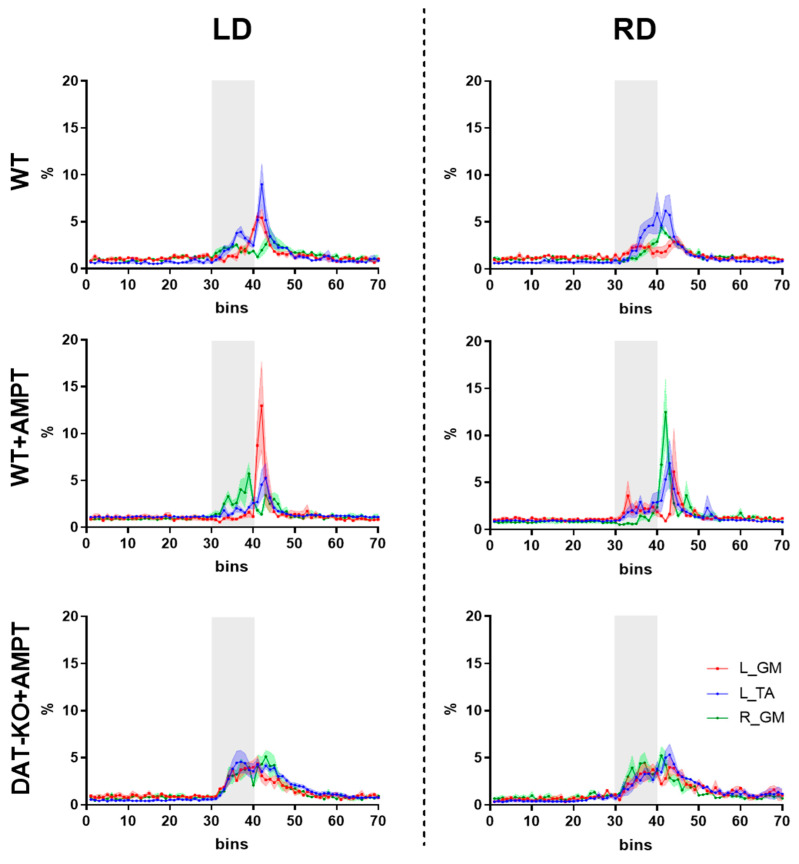
Postural correction muscle responses in rats with moderate and severe DA deficiency to the horizontal lateral leftward (LD) and rightward (RD) displacements of the supporting platform. L_TA—left hindlimb tibialis anterior muscle, L_GM (left), and R_GM (right) gastrocnemius medialis muscles. The grey area corresponded to the lateral displacement (from 30 to 40 bins). Rectified EMG signals normalized by integral muscle activity over 70 equal bins are presented as the mean ± SEM.

Analysis of the lateral horizontal displacements was carried out separately for bins 30–39 (early) and 40–49 (late), and in these time ranges, muscle activity was distributed normally (Kolmogorov–Smirnov test, *p* < 0.05). Such a biphasic pattern of muscle responses was mostly pronounced for the ipsilateral TA and contralateral GM, relative to the direction of the displacement in WT rats (Figure 3). Both early and late responses to the rightward (RD) or leftward (LD) displacements were not significantly different in comparison to normalized EMG of L_TA and L_GM in WT rats (Table 1).

### 3.3. Alteration of Postural Abilities of Rats with Moderate and Severe DA Deficiency

**Figure 4:**
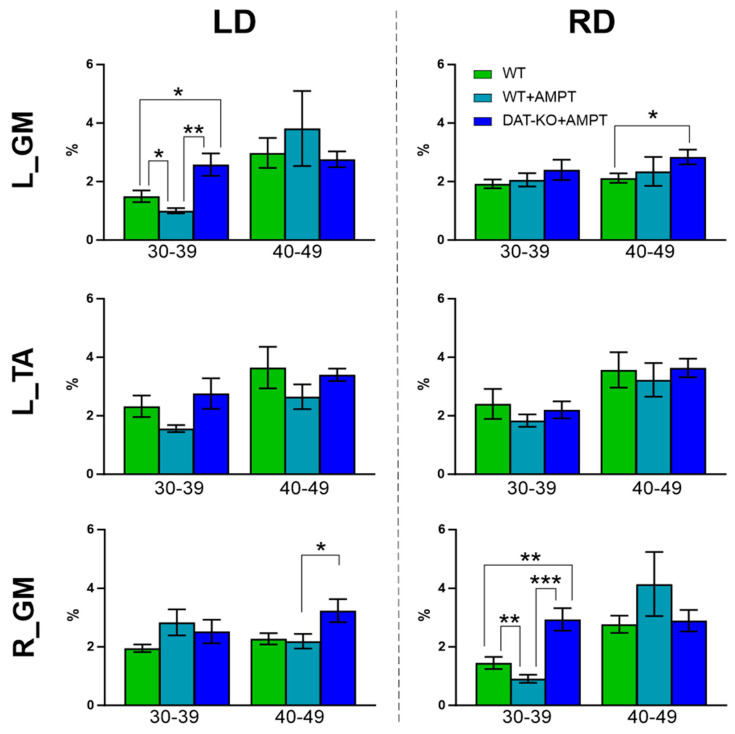
Postural correction responses of rats with normal DA level (WT), mild DA deficiency (WT + AMPT), and severe DA deficiency (DAT-KO + AMPT). L_TA—left hindlimb tibialis anterior muscle, L_GM (left) and R_GM (right) gastrocnemius medialis muscles to displacements to the left (LD) and to the right (RD) side. Data are presented as normalized by sum muscle activity. Significant differences between WT + AMPT and DAT-KO + AMPT in comparison by One-way ANOVA with post hoc Bonferroni’s multiple comparison test. *n* = 4. Mean ± SEM. *—*p* < 0.05, **—*p* < 0.01, ***—*p* < 0.001.

The onset of EMG activity increase of GM and TA responses to LD and RD was almost the same and had no significant differences between groups (Figure 3). At moderate DA deficiency (WT + AMPT) the activity of extensor (GM), ipsilateral to displacement is significantly lower than for other muscles (L_GM to LD and R_GM to RD, Figure 3 and Table 1)—the main activity delayed and occurs in the late response (40–49 bins) without any coactivation. In the early response, these rats also showed a decrease in the EMG of GM ipsilateral to the displacement compared to other groups (Figure 4 and Table 2).

Severe depletion of DA (DAT-KO + AMPT) led to the same onset of coactive EMG activity of both GM and TA (either to LD and RD, Figure 3) in early response, while in late response coactivation was less, and the differences were observed (Table 1). Relative to the WT and WT + AMPT groups, a higher activity of the extensor, contralateral to displacement, is in late response, while to extensor, ipsilateral to displacement, appeared in early response (Figure 4 and Table 2).

## 4. Discussion

The data obtained on the rats with different levels of DA depletion indicate general sensorimotor impairments and specific alteration of postural reactions during standing.

### 4.1. The Experimental Model of Progressing DA Depletion

The most common methods used in animals to investigate the mechanisms and therapy of DA-related disorders are neurotoxins and DA depletors [8]. An acute dose of neurotoxin MPTP causes blocking of the mitochondrial complex, increases oxidative stress, and eventually causes cell death and neuroinflammation. Neurodegeneration occurs within hours and stabilizes within a few days [20], and induced motor deficits can be abolished during that time [21]. Multiple injections of low doses cause symptom development over several months, and then they are stable for another few months [22]. In other words, the usage of this drug imposes time limitations both on the stabilization of the model and on the possible duration of the study with unpredictable variations. As for another commonly used neurotoxin, unilateral 6-OHDA injections lead to asymmetric motor deficits and rotational behavior [23], while bilateral injections often cause adipsia, aphagia, seizures, and high mortality [24]. 

Concerning the DA depletors—these reduce the released and circulating amount of DA in two ways [25]. The first is by blocking vesicular monoamine transporters by reserpine or tetrabenazine, which have multiple side effects associated with systemic action and influence on the release of histamine, norepinephrine, and serotonin. The second is by inhibition of the production by tyrosine hydroxylase (TH) by AMPT. It is known that TH is a necessary step in the conversion of tyrosine to levodopa, DA, and norepinephrine. It was shown that AMPT administration in rats causes a reduction in DA level and norepinephrine in normal rats with equal dynamics during the first hour [26]. However, DA in DAT-KO animals totally depende on ongoing synthesis and administration of AMPT causes dramatically depletion of DA level, while the level of norepinephrine remains at least 75% of the controls and did not differ from WT mice [12]. 

We suggest that the approach of DAT-KO + AMPT used here may be useful for modeling hypodopaminergic conditions such as Parkinson’s disease, dystonia, and parkinsonism. We observed the rapid development of motor deficits: already in 10 min after AMPT injection, the DAT-KO rats had a disturbance of the movement initiation and in 40 min—tremor and akinesia—and this condition continued for a few hours, after which mobility was recovered. Thus, DAT-KO + AMPT is a convenient, fast, reversible model of the progressive development of DA-related diseases, which may be used to study the mechanisms of movement disorders, as well as the development of therapy methods and their approbation, in particular, DBS, SCS, electrostimulation and pharmacological methods of neuromodulation.

### 4.2. The Role of Dopamine in the Control of Sensorimotor Behavior

DA is the critical neurotransmitter for locomotor activity and body postural configuration [27,28]. The main source of DA is the substantia nigra pars compacta, and DA influence on sensorimotor function is usually considered through the nigrostriatal pathway and basal ganglia modulation. However, in recent years, evidence has emerged on the direct influence of DA on the motor cortex [29] and spinal cord [30,31,32]. A lack of DA leads to disturbance in movement initiation in humans and rodents [8,20,22]. 

The noted rapid development of a severe akinetic phenotype in DAT-KO rats after the administration of the TH inhibitor AMPT, due to the selective depletion of DA in the DA-ergic terminals of DAT-KO animals, supported earlier observations in DAT-KO mice [12] and rats [13,14]. Furthermore, we observed changes in the body’s postural configuration after the AMPT injection. The pelvis is lowered in DAT-KO + AMPT rats, which is apparently due to an altered tone in the extensors. Previously, it was shown that extensor activity is more susceptible to change in rats [33] and in humans [34] with DA deficiency. It has been also demonstrated that in spinalized mice and rats, the administration of a D1 agonist potentiated the activity of the extensors [31] and induced flexion and extension rhythmic movement [30]. However, D2 agonists facilitated flexion, decreased extension, and stabilized gait [31]. Nevertheless, it should be noted that DA receptor agonists may also have a non-dopaminergic mechanism of action (an influence on serotonergic, adrenergic structures, as well as a number of other side effects). In addition, DAT-KO + AMPT put the hindlimbs wider than rats with normal DA levels, which is consistent with data observed in rats with dopaminergic cell depletion [35] and human PD subjects [36].

### 4.3. Specific Alteration of Postural Reactions after Mild and Severe DA Depletion

DA is important for maintaining balance and postural reactions [27,37]. Tests, which are usually performed for postural assessments, may be static when using low-amplitude motor tasks [38], or dynamic, elicited by surface displacements and tilts or forces applied to the body [2,39,40]. Lateral pushes or displacements are widely used to investigate postural control in different animal models [39,41,42,43,44], and rat is among the simplest mammalian models for studying postural neuronal mechanisms [45]. The lateral horizontal displacement used in our study is a dynamic evaluation that we considered more appropriate for quadrupedal animals than backward platform disturbance used in human research. 

Obviously, the muscle activity in response to perturbation depends on the experimental design (walking, standing, type of perturbation) and on the animal species. For example, in the standing cat, decreased activity was observed in GM, which was opposite to the direction of the displacement [39,43]. The muscle responses to displacement in our study consisted of two phases, as in cats [39,43] and mice [44]: the early response is due to the actual acceleration during the platform displacement, and the late response is due to maintaining balance from the inertial force after deceleration of the displacement. We did not find a difference between the normalized EMG of L_TA compared to L_GM in WT rats in early or late responses. However, the EMG activation patterns of TA and GM were opposite to the direction of the displacement in WT rats and were similar to those in walking mice, but they had higher GM activity compared to TA [44]. 

The onset of EMG activity growth of the GM and TA responses to LD and RD was almost the same, and independent from different DA levels in all groups, which is in accordance with backward surface displacement study on patients with PD [27]. Different levels of DA deficiency lead to opposite changes in muscle activity patterns. However, differences were observed in the comparison of the activity of flexors and extensors during the early response to displacement (30–39 bins) in rats with mild DA deficiency (WT + AMPT), possibly due to a violation of the mechanisms of initiation of movements. The practical lack of DA results in simultaneous and similar responses of all analyzed muscles in both phases (early and late) may be due to the pronounced hypokinetic–rigid syndrome and muscle coactivation, which is in line with reports about earlier and larger simultaneous antagonistic muscular activation in PD than in control subjects [46]. Such coactivation may serve to defend against potential perturbations when regaining equilibrium, but are not immediately available due to stiffening, meaning they cannot produce a directional response to return the body to equilibrium [46,47]. Thus, the hypodopamine state causes different changes depending on the severity and DA controls dynamic balance in rats. 

We suggest that DAT-KO + AMPT rats have disturbances in initiating postural adjustments, as was shown in rats with unilateral DA depletions: their impaired limbs produced normal supporting reactions but did not initiate correction of posture [48]. The greatest differences between the groups with altered DA levels were noted for the extensors—the L_GM to the LD, and the R_GM—to the RD, respectively. The maty be several probable explanations for this observation: DA control influences the extensors to a greater extent [30,31,33,34], or extensors can play a major role in posture corrections [44,45,49], or both. The specific role of DA in postural reactions during standing and active movements must be further investigated. 

## 5. Conclusions

We propose that the use of DAT-KO rats with dopamine synthesis inhibitor AMPT as a model can be valuable in understanding hypodopaminergic conditions like parkinsonism. AMPT caused motor deficits, tremor and akinesia in DAT-KO rats, which lasted for a few hours before mobility was restored. Lack of DA lead to posture changes in this animals: the pelvis is lowered, the hindlimbs are wider apart. AMPT administration in WT rats (mild DA deficient) did not induced changes in posture in standing position, but the extensor muscle response to postural displacements of supported platform was delayed (for the same direction, ex. left extensor and leftward displacement). Severe DA deficient in DAT-KO rats resulted in coactive extensor (GM) and flexor (TA) early responce to displacement. We assume that the presented model of DA depletion can be used to study mechanisms of DA control in different sensorimotor behaviors and to develop new therapeutic and neurorehabilitation applications in DA-related diseases.

## Figures and Tables

**Table 1:** Comparison of muscle activity in rats with moderate and severe DA deficiency in response to the leftward (LD) and rightward (RD) displacements by one-way ANOVA with post hoc Bonferroni’s multiple comparison test. *p* values are presented. Statistical significance is indicated by a color spectrum from red (significant differences) to blue (no significant differences).

		Bins	L_TA vs. L_GM	L_TA vs. R_GM	L_GM vs. R_GM	F (2, 9)
WT	LD	30–39	0.0675	0.8207	0.3198	4.021
		40–49	0.4449	0.3208	0.8752	2.434
	RD	30–39	0.8988	0.0489 *	0.1499	4.057
		40–49	0.1239	0.2971	0.1378	4.989
WT + AMPT	LD	30–39	0.0008 *	0.0198 *	0.0021 *	19.39
		40–49	0.8662	0.834	0.7886	1.368
	RD	30–39	>0.9999	0.0003 *	0.0032 *	17.19
		40–49	0.8078	0.94	0.6801	1.508
DAT-KO + AMPT	LD	30–39	>0.9999	0.7	>0.9999	1.454
		40–49	0.0357 *	>0.9999	0.8089	2.487
	RD	30–39	0.711	0.0372 *	0.0897	7.026
		40–49	0.0116 *	0.1939	>0.9999	3.355

* significant differences.

**Table 2:** Comparison of muscle corrective responses to leftward displacement (LD) and rightward displacement (RD) between rats with different dopamine levels by one-way ANOVA with post hoc Bonferroni’s multiple comparison test. *p* values are presented. Statistical significance is indicated by a color spectrum from red (significant differences) to blue (no significant differences).

		Bins	WT vs. WT + AMT	WT vs. DAT + AMT	WT + AMPT vs. DAT-KO + AMPT	F (2, 9)
LD	L_GM	30–39	0.0155 *	0.0108 *	0.0031 *	19.05
		40–49	>0.9999	>0.9999	>0.9999	0.8075
	L_TA	30–39	0.0751	0.1264	0.0741	7.057
		40–49	0.1555	>0.9999	0.1391	2.33
	R_GM	30–39	0.2391	0.4616	0.6162	3.112
		40–49	>0.9999	0.098	0.0452 *	6.363
RD	L_GM	30–39	>0.9999	0.3009	0.8376	1.759
		40–49	>0.9999	0.0033 *	0.822	2.335
	L_TA	30–39	0.5353	>0.9999	0.4232	1.667
		40–49	>0.9999	>0.9999	0.5307	0.772
	R_GM	30–39	0.0046 *	0.0041 *	0.0005 *	29
		40–49	0.4926	>0.9999	0.5678	2.125

* significant differences.

## Data Availability

The raw data used in this study are available upon request from the corresponding author.

## Supplementary Materials

The following supporting information can be downloaded at: https://www.mdpi.com/article/10.3390/biomedicines11071958/s1, **Figure S1.** Example of EMG in standing rats: WT and WT+AMPT and DAT-KO+AMPT.
