# Supplementary material for: Alteration of Postural Reactions in Rats with Different Levels of Dopamine Depletion
Source: Biomedicines. 2023 Jul 11;11(7):1958. doi: 10.3390/biomedicines11071958 (PMC10377029; doi:10.3390/biomedicines11071958)
Supplement: Supplementary file 1 [file biomedicines-11-01958-s001.zip › biomedicines-2429734-supplementary.pdf]

Supplementary Materials

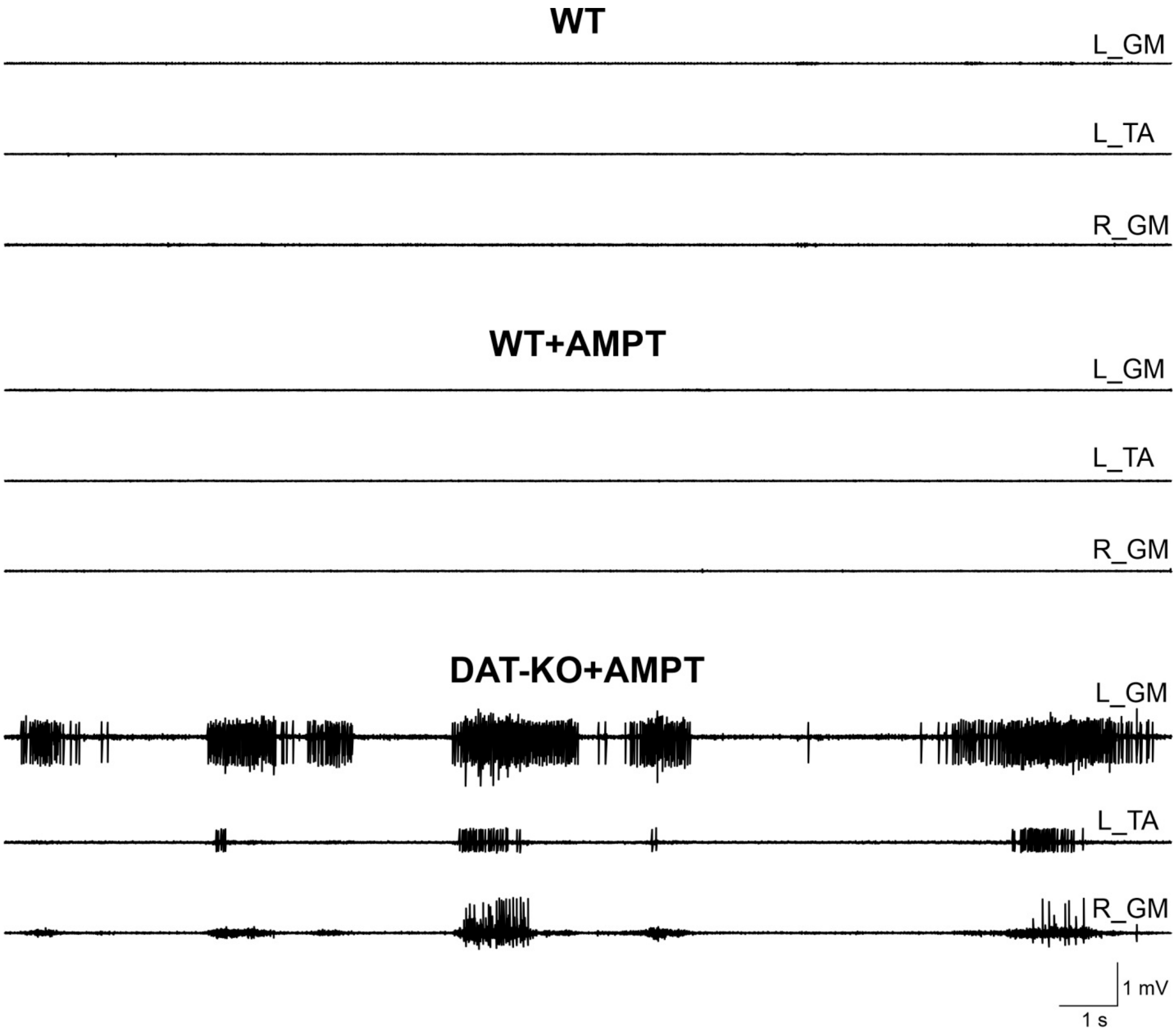

Figure S1. Example of EMG in standing rats: WT and WT+AMPT and DAT-KO+AMPT. WT and WT+AMPT had not tremor, while DAT-KO+AMPT had. EMG for left hindlimb tibialis anterior muscle (TA_L), left (GM_L) and right (GM_R) gastrocnemius medialis muscles are presented.
